# Rapid protein profiling for arthropod vector identification

**DOI:** 10.1186/1756-3305-7-S1-O11

**Published:** 2014-04-01

**Authors:** A Mathis, P Müller, V Pflüger, F Schaffner

**Affiliations:** 1 National Centre for Vector Entomology, Institute of Parasitology, University of Zürich, Zürich, Switzerland; 2 Swiss TPH, Basel, Switzerland; 3 Mabritec SA, Riehen, Switzerland

## 

Identification of insect vectors is primarily carried out using morphological features, which can be a time-consuming and difficult task. PCR-based approaches have been developed for the identification of a number of vector species. We have recently established matrix-assisted laser desorption/ionization time of flight mass spectrometry (MALDI-TOF MS), which has come of age for the high throughput identification of microorganisms, to identify imagos and larvae of biting midges (*Culicoides *spp.), and the technique has proven its suitability to accurately identify field-collected biting midges on a large scale. We have extended our insect vector reference database to also include culicid mosquito and phlebotomine species. Currently, biomarker mass sets are determined for the imagos of 15 phlebotomines (9 *Phlebotomus *spp., 3 *Lutzomyia *spp., 3 *Sergentomyia *spp.), as well as for both imagos and larvae of 38 Central European culicid species, including all established and presumptive Aedine invasive species such as the Asian tiger mosquito *Aedes albopictus *or the Asian bush mosquito *Ae. japonicus*. In addition, biomarker mass sets of eggs were determined for indigenous and invasive container-inhabiting Aedine mosquitoes (*Ae. aegypti*, *Ae. albopictus*, *Ae. atropalpus*, *Ae. cretinus*, *Ae. geniculatus*, *Ae. japonicus*, *Ae. koreicus*, *Ae. phoeniciae*, *Ae. triseriatus*) allowing to identify eggs collected in oviposition traps in the framework of surveillance programs. Eggs can be identified by MALDI-TOF MS either singly or in pools (demonstrated for pools of 10 eggs, but possibly also for larger ones) for those species of which at least 3 eggs are present in the sample. We are continuously expanding our database, e.g. to also include the remaining mosquito species whose eggs can be encountered in surveillance programs of container-inhabiting mosquitoes in Europe, i.e. species usually breeding in tree holes or rock pools in southern Europe (*Ae. berlandi*, *Ae. echinus*, *Ae. gilcolladoi*, *Ae. mariae*, *Ae. pulcritarsis*, *Ae. zammitii*, and *Orthopodomyia pulcripalpis*). Taken together, protein profiling by MALDI-TOF MS is a quick and inexpensive tool to accurately identify adult and immature stages of insect vectors collected in the field, and the technique has the potential to become the method of choice for a centralized, robust and high throughput screening of insect vector populations in connection with surveillance programs. The simple sample preparation can be done in peripheral laboratories and the slides be sent to the measuring laboratory. The future application of the method will include the accomplishment of the measurement with a mass spectrometry device anywhere and the identification via our online platform.

